# A systematic review of strategies to recruit and retain primary care doctors

**DOI:** 10.1186/s12913-016-1370-1

**Published:** 2016-04-12

**Authors:** Puja Verma, John A. Ford, Arabella Stuart, Amanda Howe, Sam Everington, Nicholas Steel

**Affiliations:** Faculty of Medicine and Health Sciences, Norwich Medical School, University of East Anglia, Chancellors Drive, Norwich, NR4 7TJ UK; NHS Tower Hamlets CCG, Alderney Building, Mile End Hospital, London, E1 4DG UK

**Keywords:** Primary care, Recruitment, Retention, Workforce, Systematic review

## Abstract

**Background:**

There is a workforce crisis in primary care. Previous research has looked at the reasons underlying recruitment and retention problems, but little research has looked at what works to improve recruitment and retention. The aim of this systematic review is to evaluate interventions and strategies used to recruit and retain primary care doctors internationally.

**Methods:**

A systematic review was undertaken. MEDLINE, EMBASE, CENTRAL and grey literature were searched from inception to January 2015. Articles assessing interventions aimed at recruiting or retaining doctors in high income countries, applicable to primary care doctors were included. No restrictions on language or year of publication. The first author screened all titles and abstracts and a second author screened 20 %. Data extraction was carried out by one author and checked by a second. Meta-analysis was not possible due to heterogeneity.

**Results:**

Fifty-one studies assessing 42 interventions were retrieved. Interventions were categorised into thirteen groups: financial incentives (*n* = 11), recruiting rural students (*n* = 6), international recruitment (*n* = 4), rural or primary care focused undergraduate placements (*n* = 3), rural or underserved postgraduate training (*n* = 3), well-being or peer support initiatives (*n* = 3), marketing (*n* = 2), mixed interventions (*n* = 5), support for professional development or research (*n* = 5), retainer schemes (*n* = 4), re-entry schemes (*n* = 1), specialised recruiters or case managers (*n* = 2) and delayed partnerships (*n* = 2).

Studies were of low methodological quality with no RCTs and only 15 studies with a comparison group. Weak evidence supported the use of postgraduate placements in underserved areas, undergraduate rural placements and recruiting students to medical school from rural areas. There was mixed evidence about financial incentives. A marketing campaign was associated with lower recruitment.

**Conclusions:**

This is the first systematic review of interventions to improve recruitment and retention of primary care doctors. Although the evidence base for recruiting and care doctors is weak and more high quality research is needed, this review found evidence to support undergraduate and postgraduate placements in underserved areas, and selective recruitment of medical students. Other initiatives covered may have potential to improve recruitment and retention of primary care practitioners, but their effectiveness has not been established.

## Background

The World Health Organisation (WHO) describes a Global Health Workforce Crisis [1, 2] with many low and high income counties experiencing difficulty recruiting and retaining doctors in rural and underserved areas [2]. The WHO has described how increased availability of healthcare workers in these areas is crucial to population health [1]. The reasons behind the workforce crisis are multifactorial but aging and expanding populations and new health challenges mean that access to good quality primary care is now more important than ever [3]. In the United Kingdom (UK) the General Practice (GP) workforce crisis is having a direct effect on patient care, deprived and rural areas are particular vulnerable [4]. There are insufficient doctors to meet demands consequently up to 543 GP practices could be forced to close within the next year [5]. The Royal College of General Practitioners (RCGP) estimated that 8000 more GPs are needed by 2020 [5] leading to the introduction of a UK target of 50 % of foundation trainees entering general practice by 2016 [6]. In the mid-1980s, general practice was the most popular career choice for medical students [7], but more recently it has become less popular than hospital medicine, with some students using it as a ‘failsafe’ or ‘backup’ career choice [8] with national applications for GP training decreasing by 6.2 % in 2015 than in the previous year [9].

Morale amongst practising primary care doctors is lower than in any other medical speciality and more are facing burnout and stress due to increasing workload and limited resources [10]. For example, in the UK 30 % of all GPs intend to leave direct patient care in the next five years [11]. The demand for part-time or flexible working patterns has increased, partly due to the increasing number of female medical practitioners, and partly due to changing expectations of both men and women [12]. Many high income countries have traditionally relied on international medical graduates (IMGs) to fill vacancies, but that is no longer possible. Despite more European-trained doctors working in the UK, the overall number of IMGs has dropped. The number of newly registered Indian doctors fell from 3640 in 2004 to 340 in 2013 [13], and 16% of overseas-qualified (outside of the European Economic Area) GPs are expected to retire over the next five to ten years [14].

Many international policies have attempted to address the problem of primary care doctor recruitment and retention [4, 15], and it is clear from other countries that significant change in a sector of a health service “requires a solid blueprint, pilot testing and evidence generation, a long-term vision, and sustained financial and political commitments“[16]. The international literature also demonstrates that it may be necessary to change the business model and professional culture in order to stabilise workforce and improve morale [17]. In the UK, £10 million has been committed by NHS England to implement a strategic ten point GP workforce action plan in 2015 in the UK [15]. Its three main areas for improvement are in recruitment, retention, and support for returning doctors. The recruitment initiative includes marketing campaign and recruitment video [18], a letter to all medical school graduates describing the positive aspects of a future career in general practice, and an additional year post training to recruit trainees to underserved areas. The report describes a three year scheme to offer financial incentives to trainees working in underserved areas. Furthermore, pilot ‘training hubs’ will offer inter-professional training to primary care staff, developing and extending the current skills base. The retention initiative includes retainer schemes and improved training capacity in general practice. Experienced GPs towards the end of their careers will be offered incentives to remain in practice, and opportunities to develop a portfolio career. Innovative ways to manage GP workload are proposed such as using physician associates, medical assistants, clinical pharmacists, and other allied health professions. A new Health Education England induction and refresher scheme aims to support GPs who have previously practiced to return to the workforce [19].

Little research has assessed the effectiveness of recruitment and retention policies for primary care doctors. The aim of this systematic review is to evaluate interventions and strategies used to recruit and retain primary care doctors internationally.

## Methods

Electronic searches of MEDLINE, EMBASE, and CENTRAL were conducted from inception to January 2015. Search terms (in English) used in MEDLINE are shown in Appendix 1. The search strategy included MESH and free text terms in three areas: 1) primary care, 2) recruitment and retention and 3) study design. Grey literature searches were undertaken in OpenSigle, internet search engines (Google and Google Scholar) and targeted websites (RCGP, Kings Fund, Health Education England, British Medical Association and specific international websites such as The Australian Government Department of Health and The World Health Organization). Specific organisations were contacted for unpublished evaluations such as Health Education England, British Medical Association and UK Local Education and Training Boards. Reference lists of the included studies and reviews were screened.

### Inclusion and exclusion criteria

To meet the inclusion criteria, studies were required to evaluate a defined intervention aimed at recruiting or retaining doctors. Studies that included medical specialities, other than primary care were included if judged to be transferable to primary care by the three authors (PV, NS and JF). Only articles from high income countries (as defined by the Organisation for Economic Co-operation and Development) were included, as the issues and strategies for low and middle income countries were very different and were mainly focused on medical migration. There was no limit on study design, language or follow-up period. Studies of other health professionals, such as nurses, were excluded. All studies without a specific intervention were excluded,

### Screening and data extraction

Titles and abstracts were screened by one reviewer (PV) and a second reviewer (JF) screened 20 % of the total sample retrieved from the search strategy to check for concordance and to minimize bias. Unclear studies were resolved through a three way discussion (PV, NS, JF). Data extracted included study details, inclusion/exclusion criteria, study design, intervention specification costs and outcome measure. Data were extracted by one author (PV) and double checked by a second (AS).

The primary outcome was number of primary care doctors recruited or retained. Secondary outcomes were recorded when available in the included studies, these were average duration of employment after recruitment, future intentions and cost. Authors were contacted for supplementary data if necessary. The risk of bias of each study was assessed by two reviewers (PV and AS) using the Newcastle Ottowa Scale, which was modified to meet the needs of the included studies to include presence of a comparison group, generalisability, conflicts of interest and quality of reporting [20]. Studies were considered for meta-analysis, but were judged to be too heterogeneous.

## Results

**Fig. 1:**
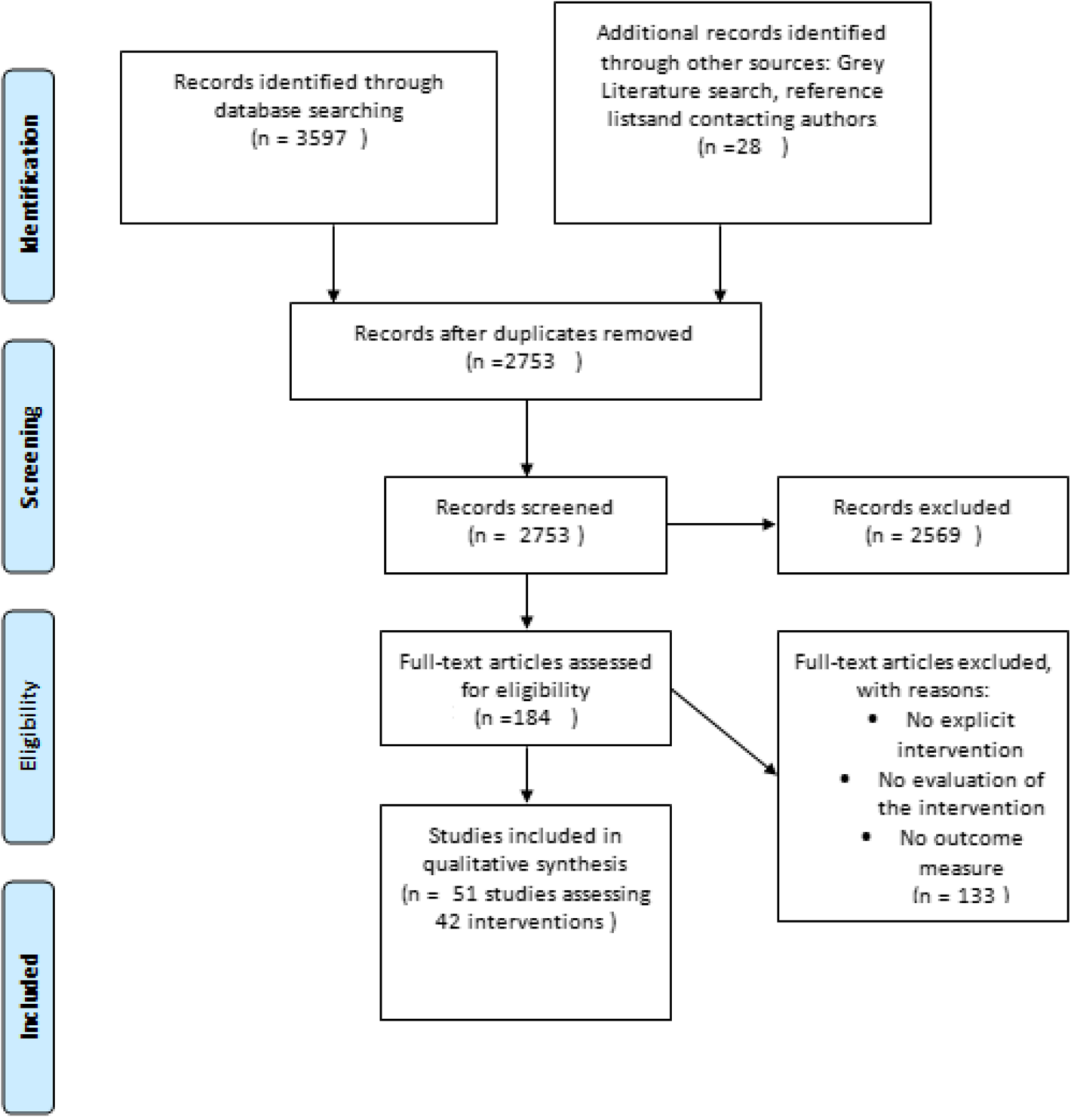
PRISMA Diagram

Three thousand five hundred ninety seven reports were identified from the electronic search (2753 after removal of duplicates) (Fig. 1) plus 28 from the grey literature search. After screening and eligibility assessment, 51 studies were included, describing 42 interventions. Seventeen studies were from the USA, twelve from the UK, eight from Australia, five from Canada, four from Norway, two from both Japan and New Zealand and one from Chile (Table 1)*.*

**Table 1:** Description of intervention, costing and outcomes

Study, (year) location		Study type sample size	Description of intervention (year)	Costs	Effect of intervention on recruitment and retention
Financial initiatives					
1	A contract-based training system for rural physicians: follow-up of Jichi Medical University graduates (1978–2006) Matsumoto et al. (2008) [21] Japan Long term effect of the Home prefecture Recruiting Scheme of Jichi Medical University, Matsumoto et al. (2008) [22] Japan	Longitudinal follow up comparative between groups = 2988 Longitudinal follow up comparative between groups *n* = 1255	Aims to recruit rural doctors and distribute them nation-wide. Jichi Medical University (1972- onwards) mission to produce rural doctors. “Home-prefecture recruiting scheme”. 9 year obligation service, 6 of those in rural practice after graduating – in their home prefecture. In exchange all undergraduate fees are waived.	If in breach of contract – all medical school expenses must be repaid in a lump sum (US $183,333), plus 10 % per year in interest.	By 2004 JMU graduates (post obligation) were 4.2 times more likely than non JMU grads to work in rural areas- • In 1994 post obligation JMU Graduates were 3.9 times more likely to work in rural areas than non JMU graduates. • Rural upbringing and primary care speciality were positively associated with ‘having a rural address at least 1 year post obligation phase. • 69.8 % of JMU graduates settled in their home prefecture after the obligation period • The rates varied from 45.5 to 93.3 % amongst the prefectures (*p* < 0.001) • Prefectures with relative shortage of physicians had higher settlement rates.
2	US department of Health and Human Services: The National Health Service Corps (NHSC) (2012) [23] (Webpage) USA The Comparative Retention of National Health Service Corps and Other Rural Physicians Results of a 9 year follow up study Pathman (1992) [24] USA The National Health Service Corps: Rural Physician Service and retention Cullen et al. (1997) [25] USA	Longitudinal follow up non comparative *n* = not reported Longitudinal follow up comparative between groups *n* = 304 Cross sectional - non comparative *n* = 2903	The National Health Service Corps (NHSC) (1970 onwards) 3 financial incentives:* Loan Repayment*: Up to $50,000 to repay their health profession student loans in exchange for a two-year commitment to work at an approved NHSC site in a high-need, underserved area. *Scholarships*: pays tuition fees, other educational costs, and provides a living stipend in return for a commitment to work at least 2 years at an approved outpatient facility in a medically underserved community. The scholarship can be awarded for up 4 years. Service begins upon graduation. *Students to Service Program Loan Repayment Program * (S2S LRP) provides up to $120,000 to medical students in their final year of school in return for a commitment to provide primary health care full time for at least 3 years at an approved NHSC site in a Health Professional Shortage Area of greatest need. NB length of obligations changed throughout the program history	Loan repayment up to $50,000 Bond of $120,000	Evaluation 1 from 2012: • 82 % of NHSC ‘clinicians continued to practice in the short term; (1 years after duration) 55 % continue to practice in underserved area 10 years after completing their service commitment. (does not specify by which specific programme they were involved in) • Primary Care Physicians who completed their NHSC commitment >10 years ago had a 60 % retention rate. Evaluation 2 from 1992 • Poor retention at 8 years employment retention rates for NHSC VS NON NHSC 29 % 52 % in rural areas (*p* < 0.001) • Hazard ratio for risk of leaving rural practice altogether was 1.74 (95 % CI 1.43-2.11). • 7/93 (7.5 %) of NHSC physicians re-enlisted in the NHS following their initial terms of obligation (mean additional years of service was 2.1 years) Evaluation 3 from 1997 • 69.8 % of the 2903 initially assigned to a rural area who entered program before 1975 had a urban practice address in 1991 27.2 % were located in their initial assignment counties.
3	Voluntary Bonding Scheme New Zealand Ministry of Health (2012) [26] (webpage) New Zealand	Cross sectional - non comparative *n* = 115	For postgraduate doctors intending to train as GPs (2009- present) or allied health professionals who are prepared to train in rural or provincial area can enter the scheme when they enter vocational training. Incentive scheme with no upfront bonding agreement to sign – after being accepted you begin working or continue to work in an eligible hard to staff community specialty or profession. If you abide by the terms and conditions of your intake year, for at least 3 years you are eligible to apply for payments after year 3, 4 and 5 years. $10,000 annual after tax payment for up to 5 years.	Incentive of $10,000	In 2009 115 doctors entered. • By 2012 102 (89 %) had opted out of the scheme. • In a survey of the 2009 cohort showed 9/37 (35 %); stated they planned to work in a hard to staff location in both the short and long term. • Significant attrition; no penalty for opting out.
4	Postgraduate medical placements in rural areas: their impact on the rural medical workforce. Dunbabin (2006) [27] Australia	Longitudinal follow up non comparative *n* = 82	Cadetship Program (1988 onwards) offering bonded scholarships to provide financial support for medical students (residents of NSW and from 2005 the Australian Capital territory) during their final 2 years of undergraduate study. In return cadets are contracted to complete 2 of their first 3 postgraduate years in the NSW rural hospital network.	Bond amount not reported	• 33/77 (43 %) of cadets entering the program before 1999 were working in rural locations in 2004 (compared with 20.5 % of medical practitioners nationally) • 17/22 (77 %) GPs were in a practice location closely related to where they completed rural service. • 44 % had chosen to specialise in GP and made up 70 % of those working in rural areas in 2004
5	A Comparative assessment of West Virginias Financial Incentive Programs for Rural Physicians Jackson et al. (2003) [28] USA	Cross Sectional - comparative between groups *n* = 251	West Virginias 4x financial incentive programs (1991 onwards): Community Scholarship Program (CSP) Average scholarship = $42,500 for students from a Health professional shortage area (HPSA) to commit to go back and serve 1 year for every year of funding received back in their home HPSA. Health Science Scholarship Program (HSSP) for fourth year medical students. $20,000 one-time award for a minimum of 2 years’ service in an underserved area. Recruitment and Retention Community Project (RRCP) : for medical residents up to $20,000 each year for up to 6 years (one year service required for each year of funding). State loan repayment program for physicians up to $40,000 for 2 year commitment contract (may be extended for 2 additional years at $25,000 a year), for minimum 2 years’ service at a non-profit site in a HPSA. Must repay the funding back in full if they default.	See individual programme	After obligations were completed – *n* = 14 (32 %) of all obligated physicians reported that they were no longer at their first service rural practice site compared to *n* = 41 (38 %) of the comparison group. (Similar retention patterns). • Obligated physicians who remained in their initial rural practice anticipated to remain an average of 18 more years in rural practice. Non-obligated physicians had similar expectations. • 6/14 (42 %) of the obligated respondents left their practice for another West Virginia rural site compared to 34/41 (82 %) of the comparison group
6	Evaluation of Physician Return for service Agreement in Newfoundland and Labrador Mathews et al. (2013) [29] USA	Longitudinal follow up comparative between groups *n* = 134	Special funded residency positions – administered by Memorial University of Newfoundland-(1997–2006) Offers funding to medical students and to postgraduate residents training in family medicine and other specialist programmes in which physician shortages were identified (the funding is gained in return for service). A Family Medicine bursary is also available to 3^rd^ and 4^th^ year medical students intending to pursue family medicine Those who accept the funding are expected to work in Newfoundland for 1 year for each year of funding received. They can also pay the money back (with interest).	Not reported	• Retention of Return For Service vs Non Return For Service physicians who first started practice between 2000 and 2005 • 11/60 (19 %) of Return For Service graduates had left the province, compared to 28/67 (42 %) of non-Return For Service graduates • RFS physicians were 3.2 times less likely compared to Non Return For Service physicians to leave the province.
7	Evaluation of the Arizona Medical student Exchange Program. Navin TR and Nichols AW (1977) [30] USA	Cross sectional - non comparative *n* = unclear	Students from 11 states which lack training facilities are given financial assistance to attend graduate programs in the health sciences.(1969-onwards) The cost that an Arizona student faces in attending an out of state medical school is covered ($500 in 1953) for return of service to Arizona: 2 years’ service for every year of participation in the program. (Reduced to 1:1 years in 1958 due to low uptake) The accepting school is also offered an ‘additional sum’ of $6000 as an inducement to accept more Arizona students in the future. The students were given the option to repay the debt in cash	Sending state paid receiving school $6000	In 1953 the first medical students were assisted by the program: • From 1953 to 1967 the program failed to the raise the number of medical school applications from Arizona. • Between 1955 and 1965 there was a consistent decline in the physician- population ratio (NB time lag whilst students are at medical school) Out of 149 program graduates • 21/149 (14 %) chose service repayment in a rural area within Arizona. Out of the 143 who have started discharging their obligation • 55/143 (38 %) chose cash repayment instead of service. • 67/143 (47 %) chose service in a metropolitan area within Arizona As of 1975 • 62 % of participants repaid there loan obligation through service in the state- but not specifically to rural areas.
8	Outcomes of states' scholarship, loan repayment, and related programs for physicians Pathman, et al. (2004) [31] USA	Cross sectional - comparative between groups *n* = 1157	5 Program types which were operating in 1996 (onwards) were compared : Scholarships- obligate medical students early in their training many years before they serve their obligations, firmly expected to provide service, hefty penalties are used to discourage them from buying out of the obligation Service option loans- targeted to medical students – can perform a service or repay the loans at standard interest rates Loan repayment – commit physicians later, nearing the end of residency. Provide assistance to repay loans accrued earlier in medical school. Minimal penalties on physicians who fail to provide a period of service. Direct financial incentives: “golden hello” to work in rural area; usually no penalty or minimal penalty for failure to complete minimal service. Resident support – financial assistance; scholarships, loan repayment and direct financial incentive- service begins 1–2 years after commitment at the end of residency.	Not reported	Obligated physicians remained longer in their Practices than non-obligated physicians (*p* = 0.03) 71 % vs 61 % at 4 years and 55 % vs 52 % at 8 years • Obligated physicians worked in underserved areas (low physician to population ratio) more often than non-obligated physicians 37 % vs 11 % *p* < 0.001 • Obligated physicians remained longer in their service practices than non-obligated physicians in their first jobs after training (HR for leaving 0.70; 95 % CI 0.51-0.96 *P* = 0.029) • Longest group retention was seen for loan repayment scheme .66 % of whom remained in their service sites for 8 years. • Service option loans reported the lowest average service completion rate (44.7 %) • Scholarship programs had a service completion rate of (66.5 %) • The highest buy out rate was for service option loan programs (49.2 %) and scholarship program (27.2 %)
Recruit rural students					
9	Long Term Retention of Graduates from a program to increase the Supply of rural Family Physicians Rabinowitz (2005) [32] USA	Cross sectional - comparative between groups *n* = 1937	The Physician Shortage Area Programme (PSAP) (1974 onwards). Recruited applicants with a rural background, eligible for financial aid (payable loans). Undertake rural family medicine placements in rural areas in their 3^rd^ and 4^th^ years.	Not reported	1937 Jefferson graduates from classes of 1978–1986 (148 PSAP graduates) 38 PSAP graduates identified. • After 11–16 years 26/38 (68 %) PSAP graduates were still practicing family medicine in the same rural area compared to 25/54 (46 %) of their non-programme peers (*p* = 0.03) in 2002
10	The Contribution of Memorial University’s medical school to rural physician supply Mathews (2008) [33] Canada	Cross sectional non comparative *n* = 1322	Long standing ‘med quest’ program (1973 onwards) to encourage secondary school students to a heath professional career. More than 30 % of memorial medical students are from rural origin compared with 1 % of other Canadian medical schools Medical school tuition is half the Canadian average	Not reported	Practice locations in 2004 were determined for graduates from 1973 to 1998. • In 2004 81/1322 (6.1 %) of graduates were working in a rural community in Newfoundland making up (20.8 %) of the rural physicians in the province. • *n* = 167 (12.6 %) graduates worked in Rural Canada making up 4.9 % of the rural physicians in Canada. • Predictors of primary care doctors working in the area included having a rural background (OR 2.52 95 % CI 1.72-3.71) being from the area (OR 5.90, 95 % 1.80-19.36).
11	Influencing residency choice and practice location through a longitudinal rural pipeline program Quinn (2011) [34] USA	Cross sectional non comparative *n* = 1046	The Missouri University Rural Track Pipeline Program (MU RTPP) (1995 onwards) has a preadmission program for rural students (rural scholars). Summer community programs for second year students: students participate in a clinical program in a rural community setting; participating hospital or clinic sponsors students and the student receives a stipend ($1000 –$2000). Aim to increase knowledge of rural medicine, improve clinical skills Six month Rural Track Clerkship (RTC) for third year medical students: students live and work in a rural community Rural track elective for 4th year medical students- one month primary care or specialty electives in a rural setting	Not reported	48 rural scholars were tracked from 2002 and compared to non-participants and RTC participants • 18/20 (90 %) of rural scholars are practicing in Missouri • 37/75 (49.3 %) of RTC are practicing in Missouri. • 57.4 % of students who participated in the RTC program chose a rural location for their first practice • Rural Scholars more than twice as likely to ‘match’ into family medicine
12	Improving the recruitment and retention of doctors by training medical students locally Landry et al. (2011) [35] Canada	Cross sectional non comparative *n* = 390	New Brunswick does not have a medical school. It’s the only Canadian bilingual province. Places reserved for New Brunswick (NB) residents in three French medical schools in Quebec since 1967 students may also undertake part of their training in their home province, and opportunity to study in first language within home province provided since 2006.	Not reported	Odds Ratios for current practice in NB by exposure to the province during training, stratified by year of undergraduate training • 4^th^ Year OR 9.3 (95 % CI 1.4-60.) • 3^rd^ Year OR 9.3 (95 % CI 1.5 -56.9) • 184/263 (70 %) of all graduates were currently practicing medicine in New Brunswick
13	Rural doctor recruitment: does medical education in rural districts recruit doctors to rural areas? Magnus et al. (1993) [36] Norway	Cross sectional non comparative *n* = 417	Established a medical school in northern Norway (1972) with the hypothesis of ‘homecoming salmon.’ Educating young people from the rural areas of northern Norway are likely to stay in these remote areas.	Not reported	Questionnaire sent to all graduates from 1979 to 1989 234/417 (56.1 %) of The University of Tromso graduates are retained in Northern Norway. • *n* = 192 (82 %) of these doctors were brought up in northern Norway.
14	Illinois RMED: A Comprehensive Program to Improve the Supply of Rural Family Physicians Stearns et al. (2000) [37] USA	Cross sectional non comparative *n* = 39	Rural Medical Education (RMED) (1993 onwards): longitudinal, multi-dimensional program with a focus on family practice. RMED provides a focused curriculum for 4 years focusing on family medicine, rural health issues and community based medicine peer support, a 16 week rural preceptorship. Students recruited – must demonstrate an orientation towards rural practice and family practice Students sign a pledge promising to complete the 4 year rural curriculum (no obligation/)	Not reported	After 6 years 39 physicians have graduated • 27/39 (69 %) of RMED alumni are in family practice residencies • 32/39 (82 %) are working in primary care
International recruitment					
15	From Spain to County Durham: experience of cross cultural general practice recruitment Bregazzi et al.(2005) [38] UK	Cross sectional - non comparative *n* = 7	‘The Durham Initiative’ Spanish General Practitioners (2002–2003) were recruited to under-doctored areas in Durham. They undertook a 4 month induction program of language training, supervised learning in the GP training environment. After induction they began their first post, continuing to meet weekly for peer group sessions facilitated by a GP trainer + Spanish born GP.	Not reported	Of the 7 GPs recruited (1 dropped out part way through the year) • 5/7 (71 %) have continued to work beyond the initial years contract • 3/7 (43 %) expect to continuing practice for between 1 and 3 years
16	Retention of J1 Visa Waiver Program physicians in Washington States Health Professional Shortage Areas. Kahn et al. (2010) [39] USA The Effect of the Physician J-1 Visa Waiver on Rural Wisconsin Crouse (2006) [40] USA	Cross sectional - non comparative *n* = 141 Cross sectional - comparative between groups *n* = 145	Conrad J-1 Visa Waiver Program: (1994 onwards) International medical graduates can agree to serve in an officially designated rural or urban underserved area in an exchange for a J-1 visa waiver; removing the usual commitment to leave the United States for a minimum of two years on completion of training. The doctors on this program are obligated to work for an approved J-1 waiver employer for the duration of their commitment period (in Washington = 3 years) J-1 Visa Waiver in Rural Wisconsin (1996 onwards) As above and commitment period (3 years).	Not reported Not reported	All J-1 Visa waiver physicians assigned to employers in Washington between 1995 and 2003 were identified 77/141 responded (55 %) • These remained with their employers a median of 23 months (0–120 months) longer than their commitment period (3 years for physicians) • 65/7 (84 %) remained with their waiver employers longer than their commitment. • 32/77 (23 %) are still working for their assigned j-1 waiver employers. • 38 % felt employers should have shown them more respect. All J-1 Visa waiver physicians assigned to employers in Wisconsin between 1996 and 2002 were identified *n* = 145, 72 responded (69 %) • 30 % of these did not complete the 3 year obligation period in the assigned community
17	Choice or chance! The influence of decentralized training on GP retention in the Bogong region of Victoria and New South Wales Robinson et al. (2013) [41] Australia	Cross sectional - non comparative *n* = 61	Decentralization of GP training (1998 onwards) to regional training providers to attract Australian born GPs + IMG’s to rural areas. Moratorium introduced in 1997 which allowed IMG and overseas born Australian trained doctor’s access to a Medicare provider number & access to government funded rebates if they trained in an accredited GP training program and practiced in ‘areas of need’ for up to 10 years. Regional training providers train GP registrars.	Not reported	• 7/26 (27 %) of the doctors subject to the moratorium who had completed their vocational training stayed in rural practice. • 24/57 (42 %) of all GPs who had completed their vocational training remained in rural general practice. 32 % (*n* = 18) remaining in Bogong region. • 73 % (*n* = 16) of the Australian born respondents and *n* = 8 (23 %) of the overseas born respondents remain in rural practice.
Rural/primary care focused placements for undergraduates					
18	Recruitment and retention of rural physicians: outcomes from the rural physician associate program of Minnesota Halaas et al. (2008) [42] USA	Cross sectional - non comparative *n* = 1175	Rural Physician Associate Programme (RPAP)(1971 onwards) 3^RD^ year medical students assigned to rural communities for 9 months. Hands on participation, one-one teaching, online curriculum participate in online discussion with fellow students meet with RPAP faculty 6 times/9 months.	Communities make a financial commitment paying $4000 to have a student for the year	Since 1971 (1175) medical students have completed the RPAP experience. • 448/901 (49.7 %) of currently practicing graduates do so in rural settings • 44 % currently practice in rural setting 100 % of the time (compared to 9 % of physicians nationally practice in rural areas) • 14 % spend 50 % of their time in a rural practice and 50 % of their time in metropolitan city • 64 % of all graduates practice in Minnesota and 36 % in rural areas of the state • 160/410 (40 %) graduates raised in metropolitan areas currently practice within rural area. • 1/2 of the RPAP class spend 2 years of medical school on a campus which actively recruits from a rural background • 896/1175 (82 %) of RPAP graduates have chosen primary care 742/1175 (68.1 %) family medicine
19	An Evaluation of the Rural Education program of the state university of New York Upstate Medical University 1990–2003 Smucny (2005) [43] USA	Cross sectional - comparative between groups *n* = 2101	Voluntary. 36 week clinical experience in rural communities for medical students that began in 1989. Until 2001 they also received a $10,000 stipend for participating in RMED. Clinical training is completed in rural communities	Until 2001 received a $10,000 stipend for participating in RMED	Between 1989 and 2003; 130 students have completed RMED: • 22/86 (26 %) of RMED programme graduates (excluding residents) practiced in rural locations vs non programme students 95/1307 (7 %) [*p* < 0.0001] • 64/76 84 % believed RMED was important in helping them choose ?A3B2 show $10#?>location
20	Geographic and Speciality Distributions of WAMI program Participants and Nonparticipants Adkins et al. (1987) [44] USA	Cross sectional - comparative between groups *n* = 2704	WAMI Program (1975 onwards) The states of, Alaska, Montana and Idaho, which lack training facilities entered into a cooperative medical education program- with The University of Washington. It would accept 20 students each from Montana and Idaho and 10 from Alaska each year. It has a decentralized medical school program where teaching occurs in rural areas.	In 1982 the programme cost the 4 states $4.8 million collectively.	Graduates from 1975 to 1981 included: In 1984: • *n* = 156/677 (23 %) of graduates with programme experience were working in a non-metropolitan area compared to *n* = 32/260 (12 %) of graduates without programme experience.
Rural/underserved postgraduate placement					
21	Where are they Now The Career paths of Remote Vocational training scheme registrars Wearne (2010) [46] Australia	Cross sectional -non comparative *n* = 24	The remote vocational- training scheme (1999–2005) trains doctors in remote communities using distance education and supervision. Standard program was 3 years duration. Contact with supervisors is minimal (a minimum of 1 h per week in the first 6 months, 1 h per fortnight in the second 6 months, and 1 h per month thereafter using telephone, text, fax, email or internet videoconferencing). Registrars attend weekly tele-tutorials and develop their clinical and procedural skills needed for the extended scope of remote clinical practice at 2 yearly face to face workshops	Not reported	• 24 doctors graduated from the training scheme • 6 graduates (25 %) work in the same location as they trained. • 17/21 (81 %) in rural areas • 20/21 (95 %) still work in Australia
22	Experiences of female General practice registrars: are Rural attachments encouraging them to stay? Charles et al. (2005) [45] Australia	Cross sectional - non comparative *n* = 83	Mandatory minimum of 6 months training in a rural area for GP registrars on the General Practice Education and Training Program (2002).	Not reported	• 21/65 (32 %) of registrars reported being more likely to work in a rural area as a direct result of the attachment • 9/65 (14 %) were influenced against it as a direct result of the attachment. • Plans to work in a rural area were positively associated with prior rural residence • Registrars on specific rural pathway training had more intention of working in a rural location after graduation 49 % compared to 22 % of general pathway registrars (*p* < 0.05)
23	Training family physicians in community health centres: a health workforce solution Morris et al. (2008) [48] USA	Cross sectional - comparative between group *n* = 1312	Community health care centres (1980 onwards) federally funded primary care clinics that provide care for underinsured and uninsured patients trained family medicine graduates– with the hope that they will be better prepared and more likely to meet the health workforce demands	Not reported	OR’s for current practice in underserved area based on training exposure: • Family physicians training in a programme OR 2.7 (95 % CI 1.6-4.7) compare to non-programme trained physicians • 63.9 % of CHC trained physicians working in underserved area compared to 37.3 % non-programme physicians.
Well-being/peer support initiatives					
24	Impact of support initiatives on retaining rural general practitioners Gardiner et al. (2006) [48] Australia	Longitudinal Study comparative (before and after) *n* = 221	The DR DOC programme introduced in 1999 (onwards) as a rural workforce support programme offering both social and emotional support strategies as well as practical interventions to help improve primary care doctors health and wellbeing including peer supported networks, emergency support lines and rural retreats, and health check-ups for rural doctors and their families.	Not reported	Followed up in 2001 (time 1) and 2003 (time 3) • Moderate reduction of 5 % of those considering on leaving rural General Practice after the Course ‘’Time 1”: 98/187 (52.7 %) to ‘’Time 2” : 102/221 (46.1 %)
25	Postgraduate training at the ends of the earth - a way to retain physicians? Straume et al. (2010) [50] Norway Internship at the ends of the earth - a way to recruit physicians? Straume et al. (2010) [49] Norway	Longitudinal study non comparative *n* = 36 Cross sectional - comparative between group *n* = 233	Special tutorial group started in 1997 (onwards) for postgraduates serving a 18 month medical internship in rural area (normal in Norwegian training program) to enhance retention, decrease professional and social isolation	Not reported	29/36 (80 %) family doctors were still working in Finnmark In 2003/4, 6 years after completing their tutorial. • Overall 5 year retention rate of 65 % • Interns bought up in the north were 8 times more likely to take their first job in the north as those from a southern background (C1 2.2-29.6) • Interns who graduated from the University of Tromso were 3.6 times more likely to take a job in the north than their counterparts from the southern universities.
Marketing					
26	The Effects of Video Advertising on Physician Recruitment to a Family Practice Residency Program Barclay (1994) [51] USA	Longitudinal follow up comparative between group *n* = 248	A promotional video (1992–1993) described the University of Maryland family practice program- highlighted intellectual challenges/scope of family practice. The video was sent to half of all persons inquiring about the residency programme. The remaining inquiries received all standard application materials and the residency brochure but not the video tape.	Not reported	120 people received the video • 35 (29 %) of those who received the video completed the application process compared to 69 (54 %) who did not receive the video • Controls (applicants who did not receive the video) were 2.86 x more likely (95 % CI 1.6- 5.0) to apply to the programme (*p* < 0.0001) • After interview 0 of the 120 persons who received the video matched with the residency program (*p* <0.005).
27	The Effect of a Blog on Recruitment to GPST in the north of Scotland Green (2015) [52] UK	Cross sectional - non comparative *n* = unclear	Online Blog (2012–2013) static pages and a dynamic blog section to feed information on GP training programme in the north of Scotland. Five existing GP trainees blogged about their experiences of GP training in the north of Scotland. Newly qualified GPs wrote articles describing their time in training and subsequent careers- to demonstrate the variety of career paths in GP.	Not reported	Survey of year 1 GP trainees in Aug 2013 and 2014, 76 % of those surveyed had viewed the blog • 48 % of those surveyed the blog had influenced the choice of location for training. • The fill rate at the end of round 1 was up to 71 % in 2013 and 81 % in 2014 from an average of 60 % in the years prior to the blog. (At this time the overall recruitment in Scotland remained static.)
Mixed approach					
28	Recruiting and Retaining GP’s to remote areas in Northern The Senja Doctor Project. Conference presentation (2010) [53] Norway	Cross sectional non comparative *n* = unclear	Project aimed to develop a collaborative model for GP services in the four Senja Municipalities (2007–2009). Establish a new main GP office where all doctors meet one a week. All doctors part of a professional network with a fixed wage Driving to local offices included in working time, organized scientific activity, no public health responsibilities and continued medical education programs	Not reported	• One municipality had recruited and lost 73 GPs in 10 years prior to this scheme • 2 years from the start of the programme in Feb 2007 4 of 10 GP applicants signed a job contract, 2 with GP specialist qualifications and 2 residents.
29	The Chilean Rural Practitioner Programme: a multidimensional strategy to attract and retain doctors in rural areas Pena (2010) [54] Bulletin of the World Health Organization Chile	Longitudinal follow up non comparative *n* = unclear	The Rural practitioner Programme launched in 1955; four domains of incentives and a competitive application process Education – voluntary rural clerkship- 4 week clerkship with physicians from the RPP Financial – direct and indirect incentives; direct = salary + tuition fees paid for increments full time compensation of 23 %, indirect e.g. installation and departure kit – double salary for 1^st^ and last month transport tickets and a removal van Management, environment and social support incentives to engage in hospital and community work, continuous professional development, increased holiday and leave allocation. External incentives – internship during medical school	Double salary for 1^st^ and last month plus travel allowance	58 % of rural practitioners are retained for the maximum period (6 years) • High degree of satisfaction with the program >90 % considered it a positive experience • Applications exceed the number of available positions by at least 2.5 times
30	Alberta Rural Physician Action Plan : an integrated approach to education, recruitment, and retention Wilson (1998) [55], Canada Rural Incentive Programs a failing report card Czapski (1998) [56] Canada	Cross sectional - non comparative *n* = unclear Cross sectional - non comparative *n* = unclear	Alberta Rural Physician Action Plan: (1991 onwards) Addresses recruiting and retaining rural physicians at the medical student, resident and current physician levels. Undergraduate medical students and residents Rural rotations Special skills program Student loan remission program Mandatory four week Family Medicine rotation (most students in rural Alberta) Physicians currently practicing in rural Alberta CME initiatives Enrichment program Rural locum program	Government provided RPAP with funding of £3.11 million per year	• 1995 - 35 % of 285 responding physicians indicated the RPAP had a critical or moderate on their decision to move to or stay in rural Alberta. • By 1998 the number of rural primary care doctors had dropped 34 % from 1994 baseline figure
31	Ontarios Underserviced Area Program Revisited: an indirect analysis Anderson et al. (1990) [57] Canada	Cross sectional - non comparative *n* = unclear	Ontarios Underserviced Area Program Started in 1969 (onwards) : To place physicians in areas on Ontario deemed to be medically underserved 36 bursaries of $5000 awarded annually to Ontario students. Students are expected to return service to an underserved area following their training (if the student fails to fulfil the service, the bursary is refundable with interest) $40,000 incentive payment paid Quarterly over 4 years for physicians	$40,000 incentive payment paid quarterly over 4 years to physicians $5000 student bursary	• Physician population ratios have improved • Each northern country experienced between 35 and 80 % improvement in its ratio between 1956 and 1986 • The province improved its ratio from 971 people/physician in 1956 to 560/physician in 1986
Support for professional development and research					
32	Developing primary care through education Hilton et al. (1997) [58] UK Academic Training in London. GP Tomorrow (book) Freeman et al. (2002) [59] UK Whole-system evaluation research of a scheme to support inner city recruitment and retention of GPs Bellman (2002) [60] UK	Cross sectional non comparative *n* = not reported Cross sectional non comparative *n* = 49 Cross sectional non comparative *n* = 14	The London Initiative Zone Educational Incentives Scheme (LIZEI) Aim of the programme was to improve recruitment, retention and refreshment of London GPs (1994–1999) London Academic Training scheme (LATS) (1995–2000): To encourage GPs to remain in the London area each with a strong link to an academic department. 7 ½ day sessions/week for academic training, 2 sessions in general practice GP Assistants/Research Associates: (time period not reported) recently vocationally trained GPs provide regular clinical cover to LIZ practitioners 7 clinical sessions + sessions for research and development.	LATS: Practice pay registrar £80 for medical defence subscription and travel LATS: Total budget in the first year for 12 registrars = £600,000	LATS: • 2 Years on: 75 % of the first cohort continue to practice in London, with academic links LATS: In 2000 the 49 participants of the first 4 cohorts were contacted with 37 replies. • 32/37 (86 %) were working in London • 34/37 (92 %) In general practice. • 20/37 (54 %) were current members of academic departments. GP Assistants/Research Associates: Participants in the 9 month scheme and a previous years scheme were contacted, • 7/14 (50 %) have become principals/partners • 10/14 (71 %) chose to remain working in local practices
33	Positive Impact of Rural Academic Family Practice on Rural Medical Recruitment and Retention in South Australia. Wilkinson et al. (2001) [61] Australia	Longitudinal follow up non comparative *n* = 17	Four rural academic family practices (1995–1999) Established with support of University of Adelaide. Doctors work for fee-for service bases (no need for financial commitment). Strong academic component- teaching students, pursuing research.	Not reported	From 1995 to 1999 • Recruitment: 17 doctors were recruited, 14 were placed in the 4 academic family practices • Retention: 4 doctors left after an average of 20 months (6 months – 3 years and 6 months) mean duration of appointment = 15 months (range = 4 months to 3 years and 6 months) • 5/17 (24 %) of the doctors were overseas trained, 4/5 expected to stay (80 %) permanently.
34	Making a difference: education and training retains and supports rural and remote doctors in Queensland. White (2007) [62] Australia	Longitudinal non comparative *n* = 426	Continuing medical education opportunities (2004–2006) were provided in the aim to retain medical practitioners in rural and remote communities. Workshops on topics such as emergency cardiology.	Travel, accommodation + locum support subsidised	341/426 (80 %) of respondents agreed or strongly agreed they were less likely to remain in rural practice without access to CME workshop
Retainer schemes					
35	The GP Retainer Scheme: report of a national survey. Lockyer et al. (2014) [65] UK	Cross sectional non comparative *n* = 318	The GP retainer scheme (1998-present) combines a service commitment and educational element. It allows a limited number of sessions in clinical practice (1–4 sessions per week, 5 years max (unless in exceptional circumstances) to aid the retention of skills when taking time out	Retainer payment £59.18 per session £310 for professional expenses	Of those who had left the scheme in the last 2 years (2012–2013) *n* = 105 were working as: • 91/105 (86.3 %) GPs, • 49/105 (47.1 %) in salaried GP posts. • 25/105 (24 %) were working as GP principals/partners • Future plans of current retainers: 93/105 (88.6 %) planned to continue as GPs.
36	Doctors' retainer scheme in Scotland: time for change? Douglas et al. (1996) [66] UK	Cross sectional; non comparative *n* = 357	Doctors' retainer scheme in Scotland (1972–1998) allows a limited number of sessions in clinical practice to aid the retention of skills when taking time out. Terms: Must subscribe to a professional journal, carry out a maximum of 2 sessions per week and at least 12 per year and attend a minimum of 7 education sessions per year.	Retainer fee of £290 + salary The practice receive a fee of £40.50 per session	Length of membership 1–17 years Former members who responded *n* = 104 • 76/104 (73 %) had left the scheme within 4 years • 31/104 (29 %) were GP principals/partners • 5/104 (4 %) were unemployed. • 33/104 (32 %) stated it prevented them from leaving medicine Of current members *n* = 152 • 69/152 (46 %) stated the scheme prevented them from leaving medicine
37	Special provisions for women doctors to train and practice medicine after graduation: a report of a survey Beaumont (1979) [63] UK Review of the women’s doctors retainer scheme in Sheffield region 1972–1973 Eskin (1974) [64] UK	Cross sectional non comparative *n* = 2433 Cross sectional non comparative *n* = 14	UK Women’s Doctors retainer scheme (1973–1976) for female doctors in hospital medicine, GP or that work in the local authority health service aged under 55; who are unemployed (or work ≤2 sessions per week) Terms: As above and membership with a medical defence organisation	£ 50 retainer fee	• 36/2433 (1.5 %) of respondents had been a member of the retainer scheme and 91 % of them were currently working; 5 (14 %) in full time posts Evaluation 2:14 doctors on the scheme • 10/14 (71 %) of these subsequently employed in the Sheffield region; (hospital doctors + GPs)
Re-entry scheme					
38	Putting principals back into practice: an evaluation of a re-entry course for vocationally trained doctors Baker et al. (1997) [67] UK	Longitudinal follow up comparative between groups *n* = 14	Re-entry course (3 day course March 1996) developed to help doctors to return to general practice. Rebuilding confidence and needs based. 8 tutorial sessions (rational prescribing, developments in therapeutics, recent advances, CPR, practice management, employment prospects) and simulated surgeries.	Charge £450 per delegate	6 months post course • 2/14 (14 %) have returned as principals/partners • 7/14 (50 %) had made ‘positive steps’ to return to general practice • Vs 1 in the control group (size of the control group not stated in text) had made plans to return to practice
Delayed partnership					
39	Career Start in County Durham Tomorrow’s GP (Book) Harrison et al. (2002) [68] UK	Cross sectional - non comparative *N* = unclear	GP Career Start Scheme: (1996) 2 year salaried GP Start Scheme ‘Give Vocationally trained practitioners a further level of training to aid the difficult transition between registrar and principal/partner + aid the personal and professional development of its doctors Year 1 sessions in mentor practices + half day release for group education Year 2 50 % general practice locums in County Durham + 50 % Professional and personal development	Full time salary at 80 % of net intended GP principal/partner income. +/− a bonus of 10 % of final salary to join Durham Medical List	Seven recruited (5 women, 2 men) in 1996 (since then 5 further cohorts have been recruited). 19 had left the scheme by 2002,Career destinations of the above 19:100 % remain working as a GP in some capacity Remaining in County Durham: • Principal/partner 6/19 (33 %), retained 3/19 (16 %), salaried post 2/19 (11 %) Working in wider NHS • Principal/partner 3/19 (16 %) retained 2/19 (11 %), Salaried Post 2/19 (11 %), Locum 1/19 (5 %)
40	South London Vocational Training Associate scheme seven years on GP Tomorrow (book) Delacourt et al. (2002) [69] UK	Cross sectional - non comparative *n* = 50	An extra structured year of professional development (1994–2002) in general practice and to also allow this time as ‘cover’ for existing practitioners at the practice. Vocational Training Associate scheme 7 sessions working in 2 busy inner city practices + research and professional development time.	Not reported	Since 1994–2002 50 GPs have been on the scheme • 7 years on 22/50 (44 %) still work as GP principals/partner/salaried doctors in the schemes locality • 7/50 (14 %) remain as assistants or locums in the schemes locality
Specialised recruiter/case manager					
41	Recruitment of rural health care providers: a regional recruiter strategy Felix et al. (2003) [70] USA	Cross sectional - non comparative *n* = 8	Delta- based recruiter (DR) (2000–2002) to assist communities with health care provider recruitment and retention of uses a holistic approach + encourages community development activities – Nurtures new providers to ease their transition into their new community	Salary and ‘fringe’ for 1 full time Delta recruiter $75,000 a year Average cost of $18, 750 per recruit	In a 2 year period • DR was able to recruit 8 primary care providers (3 primary care physicians) • Previously only had access to a part time rural health clinic managed by a nurse practitioner
42	Case management: a model for the recruitment of rural general practitioners MacIsaac, et al. (2000) [71] Australia.	Cross sectional - non comparative *n* = 17	The West Vic Model: (Feb 1997- May 1998) intensive case manager to identify potential doctors, assess any issues, define goals, support and motivate them and help ease the transition (national and international recruitment)	$1000-$1500 cost of advertising in 2 newspapers per week. Other costs not detailed.	Over 18 month period • 17 doctors placed into temporary or permanent placements • 4/17 (23 %) from UK/Ireland

The length of follow up ranged from one to 32 years. The number of participants included in the studies ranged from 7 to 2988. Sample sizes were generally small and 7 studies had less than 20 participants. In 8 studies the outcome was the self-reported location where the trainee or doctor was practicing after the intervention. Some studies used national databases or practice address as the outcome measure.

There were no randomised control trials (RCTs). 38 used a cross-sectional design, of which 30 did not include a comparison group, and 8 had a between-group comparison. Thirteen studies were a longitudinal design, of which six lacked a comparison group. Of the seven longitudinal comparison studies, one was a before and after comparison and six compared two parallel groups.

The representativeness of the included participants was generally good, however the absence of a comparison group resulted in a high risk of bias in many studies (Table 2)*.* Assessment of the outcome and follow-up was generally low risk of bias. Most studies were described in an adequate or detailed manner and had potential or good generalisability. 21/51 of the included studies had a conflict of interest; primarily the study authors who undertook and evaluated the intervention were part of the same organisation that delivered the intervention.

**Table 2:** Risk of bias table: modified Newcastle Ottowa

	Author and year	Representativeness of intervention group (selection bias)	Control group	Representativeness of control group (selection bias)	Comparability of intervention and control group	Adequate assessment of outcome	Adequate follow-up	Reporting	Generalizability	Conflicts of interest
1	Lockyer L et al., (2014) [65]	Unclear	High	N/A	N/A	Low	N/A	Detailed	Good	No
2	Douglas A & McCann I, (1996) [66]	High	High	N/A	N/A	Low	N/A	Adequate	Good	No
3	Beaumont B (1979) [63]	Unclear	High	N/A	N/A	Low	N/A	Incomplete	Potential	No
4	Eskin F, (1974) [64]	Low	High	N/A	N/A	Low	Low	Detailed	Limited	No
5	Baker M et al. (1997) [67]	Low	Low	Unclear	Low	Low	Low	Limited	Limited	No
6	Hilton S et al. (1997) [58]	N/A	N/A	N/A	N/A	High	N/A	Detailed	N/A	Yes
7	Freeman et al. (2002)	Low	High	N/A	N/A	Low	Low	Adequate	Limited	No
8	Bellman L (2002) [60]	High	High	N/A	N/A	Low	N/A	Detailed	Limited	Yes
9	Wilkinson D (2001) [61]	Low	N/A	N/A	N/A	Low	Low	Adequate	Potential	Yes
10	White CD et al. (2007) [62]	Unclear	High	N/A	N/A	Low-	N/A	Incomplete	Limited	Yes
11	Gardiner M et al. (2006) [48]	Unclear	High	N/A	N/A	High-	Unclear	Detailed	Potential	No
12	Straume et al. (2010) [50]	Low	High	N/A	N/A	Low	Low	Detailed	Potential	No
13	Straume and Shaw (2010) [49]	Low	Low	Unclear	Unclear	Low	Unclear	Detailed	Potential	No
14	Felix H et al. (2003) [70]	Unclear	High	N/A	N/A	Low	N/A	Adequate	Potential	No
15	MacIsaac et al. (2000) [71]	Unclear	High	N/A	N/A	Low	N/A	Adequate	Potential	Yes-
16	Bregazzi R and Harrison (2005) [38]	Low	High	N/A	N/A	Unclear	Unclear	Poor	Limited	No
17	Kahn TR et al. (2010) [39]	Low	High	N/A	N/A	Low	Low	Detailed	Limited	No
18	Crouse BJ and Munson RL (2006) [40]	Low	High	N/A	N/A	Low	Unclear	Adequate	Potential	No
19	Robinson M and Slaney GM (2013) [41]	High	High	N/A	N/A	Low 2X Data sets	High	Adequate	Potential	Yes
20	Rabinowitz et al. (2005) [32]	Low	Low	Unclear	Unclear	Low	Low	Adequate	Potential	Yes
21	Mathews M et al. (2008) [33]	Low	High	N/A	N/A	Low	Low-	Detailed	Potential	Yes
22	Quinn KJ et al. (2011) [34]	Low	Low	Unclear	Unclear	Low	High	Adequate	Potential	Yes
23	Landry et al. (2011) [35]	Low	Low	Unclear	Low	Low	High	Detailed	Limited	Yes
24	Magnus JH & Tollan A (1993) [36]	Low	High	N/A	N/A	High	Low	Adequate	Potential	Yes
25	Stearns et al. (2000) [37]	Low	High	N/A	N/A	Low	Unclear	Limited	Potential	Yes
26	Halaas et al. (2008) [42]	Low	High	N/A	N/A	Low-	Unclear- ‘	Detailed	Good	Yes
27	Smucny J et al. (2005) [43]	High	Low	Low	Unclear	Low	High	Adequate	Good	Yes
28	Adkins et al. (1987) [44]	Low	Low	Unclear	Unclear	Low	Low	Adequate	Potential	Yes
29	Wearne S et al. (2010) [46]	Low	High	N/A	N/A	Low	Low	Detailed	Limited	Yes
30	Charles et al. (2005) [45]	Low	High	N/A	N/A	Low	Low	Adequate	Potential	No
31	Morris CG et al. (2008) [47]	Low	Low	Low	Low	Low	Unclear	Detailed	Potential	No
32	Barclay et al. (1994) [51]	Low	Low	Unclear	Unclear	High-	Unclear	Poor	Limited	Yes
33	Paul Green (2015) [52]	Unclear	N/A	N/A	N/A	High	Low	Limited	Potential	Unclear
34	Harrison J and Redpath L (2002) [68]	High	High	N/A	N/A	Unclear	Low	Adequate	Potential	No
35	Delacourt L and Savage R (2002) [69]	Low	High	N/A	N/A	Unclear	Low	Adequate	Potential	No
36	Matsumoto M et al. (2008) [21]	Low	Low	Low	Unclear	Low	Low	Detailed	Good	No
37	Matsumoto M et al. (2008) [22]	Low	High	N/A	N/A	Low	Low	Detailed	Good	No
38	US department of Health and Human services Health resources and services administration (2012) [23]	Unclear	High	N/A	N/A	High	Low	Limited	Potential	Yes
39	Pathman D et al. (1992) [24]	Low	Low	Unclear	High	Low	Low	Detailed	Good	Yes
40	Cullen TJ et al. (1997) [25]	Low	High	N/A	N/A	Low-	Low	Detailed	Good	No
41	New Zealand Ministry of Health (2012) [26]	Unclear	High	N/A	N/A	High	Unclear	Limited	Potential	No
42	Dunbabin JS et al. (2006) [27]	High –	High	N/A	N/A	Low	Low	Adequate	Potential	Yes
43	Jackson J et al. (2003) [28]	Low	Low	Low	High	Low	Low	Adequate	Limited	NO
44	Mathews M et al. (2013) [29]	Low	Low	Low	Low	Low	Low	Adequate	Good	Yes
45	Navin TR and Nichols AW (1977) [30]	Low	High	N/A	N/A	Low	Low	Detailed	Limited	Yes
46	Pathman et al. (2004) [31]	High	Low	Low	Low	Low	Low	Adequate	Good	No
47	WONCA (2010) [53]	Unclear	High	N/A	N/A	Unclear	Unclear	Limited	Limited	No
48	Pena S et al. (2010) [54]	Unclear	High	N/A	N/A	Unclear	Unclear	Limited	Limited	No
49	Wilson et al. (1998) [55]	Unclear	High	N/A	N/A	High	Unclear	Limited	Limited	No
50	Hutten – Czapski (1998) [56]	N/A	N/A	N/A	N/A	High	Unclear	Limited	Limited	No
51	Anderson M & Rosenberg (1990) [57]	N/A	N/A	N/A	N/A	High	Low	Detailed	Limited	No

### Interventions tested

Interventions could be broadly categorised into 13 groups: retainer schemes, re-entry schemes, support for professional development or research, specialised recruiters or case managers, well-being or peer support initiatives, recruiting rural students, rural or primary care focused undergraduate placements, rural or underserved postgraduate training, marketing, delayed partnerships, international recruitment, financial incentives and mixed interventions. Results are presented from strongest to weakest evidence.

### Financial incentives

The strongest evidence was for financial incentives, eleven studies evaluated interventions which provided financial incentives in return for an obligation of service [21–31]. Six studies had a comparison group [21, 22, 24, 28, 29, 31]. Two Japanese studies examined a strategy which obligated students to a nine year service agreement in their home region in exchange for fully funded undergraduate training (medical school) [21, 22]. After the nine years, students were 4.2 times more likely (no statistical significance reported) to work in rural areas compared to non-obligated students [21]. A comparative study of financial incentives compared with no financial incentives in West Virginia found similar retention rates after the obligation period (32 % 14/44 vs 38 % 41/108), with no statistical significance reported [28]. A study of five separate financial incentives found that retention rates were statistically significantly higher for obligated than non-obligated doctors (Hazard ratio 0.70 95 % CI 0.51 to 0.96) [31]. Three studies, only one of which had a comparison, assessed the National Health Service Corps (NHSC) scheme in the USA which used financial incentives, loan repayment or scholarship throughout their medical education [23–25]. The only one of these studies with a comparison group showed that NHSC participants had a lower retention rate compared to non-NHSC participants (29 % versus 52 %, *p* value < 0.001) [24]. One study found doctors were 3.2 times less likely to leave an underserved area if they were fulfilling a service obligation in repayment for a funding during medical school or during postgraduate training [29].

The remaining studies did not have a comparison group and were therefore difficult to draw conclusions from. A postgraduate voluntary bonding scheme in New Zealand which recruited trainees to hard-to-staff communities for five years, with payments starting after the third year and no penalty for withdrawal, found that 89 % of graduates had opted out of the scheme three years after entering [26].

### Recruiting rural students

Evidence to support recruiting rural students was also found. Six studies, only one of which had a comparison group, evaluated recruiting rural students to medical school, with the expectation that some would return to their home town for practice [32–37]. The comparative study found that 68%were still practicing family medicine in the same rural area up to 16 years after graduating compared to 46 % in the comparison group (*p* = 0.03) [32]. While the remaining five studies [33–37] found that a large proportion of individuals recruited from rural areas subsequently work in rural areas (one study reported up to 90 %) [34], the lack of a comparison group makes it difficult to determine what would have happened if recruitment from rural areas had not taken place.

### International recruitment

Four studies (three without comparison groups) evaluated international recruitment schemes [38–41]. Three initiatives waived certain visa or work requirements to enable IMGs to work in USA or Australia if they agreed to work in rural or underserved areas for an obligated period of up to ten years [39–41]. The comparative study from USA found doctors recruited entering practice without J-1 Visa Waivers in rural communities had a significantly higher retention rate than their visa waiver colleagues (*p* < 0.001) [40]. These schemes recruited IMGs with varied retention rates. All studies reported success in recruiting international doctors (range 7 to 145), but the three lacking of a comparison group were difficult to draw conclusions [38–41]. Three studies found that a significant number of IMGs did not stay in rural practice (73 % 19/26) [41], did not complete the three year obligation period (30 %, 22/72) [40] or did not work beyond the initial years contract (19 %, 2/7) [38].

### Rural or primary care focused undergraduate placements (i.e. undergraduate placements refers to placements during medical school)

Three studies from the USA looked at rural undergraduate placements in primary care settings [42–44]. One comparative study found that 23 % (156/677) [44] of individuals with rural experience during their undergraduate were practicing in rural areas compared to 12 % (32/260) [44] of students without (statistical significance not reported). One study found a higher percentage of graduates with rural exposure in medical school subsequently worked in rural areas than those without (*n* = 1393, 26 % vs 7 %, *p* < 0.001) [43]. The final study reported a high proportion of students practicing primary care in rural areas after rural placements, but without a comparison group it is difficult to draw conclusions [43].

### Rural or underserved postgraduate training

Three studies evaluated postgraduate training in rural/underserved areas [45–47]. One comparative study from the USA found that doctors who were trained in a community health centre serving underserved communities were statistically significantly more likely (odds ratio 2.7, 95 % CI 1.6 to 4.7) to work in underserved areas compared to doctors who had not [47]. Two studies from Australia did not have a comparison group but one reported that a small percentage (14 %) of individuals reported that they were influenced against rural practice after their placements [45].

### Well- being or peer support initiatives

Three studies provided social and emotional support to rurally isolated doctors [48–50]. One Australian study using a before and after comparison found a moderate reduction of 5 % (98/187 compared with 102/221, statistical significance not reported) in those planning to leave rural practice after a support initiative was introduced [48]. Two studies from northern Norway reported on a tutorial group which primarily provided support for postgraduate

doctors serving an internship in a rural area [49, 50]. The authors found good recruitment (twice as many as expected) [49] and retention (65 % five year retention) [50], but the results were confounded by place of graduation and growing up in that area making it impossible to disaggregate the effects of the tutorial group.

### Marketing

Two studies evaluated marketing strategies for recruiting residents to a primary care training program [51, 52]. A promotional video marketing in the USA was associated with lower recruitment with only 29 % (35/120) of those receiving the video applied compared to 54 % (69/128) of those who did not (*p* < 0.0001) [51]. In a non-comparative study, 48 % of trainees recruited in the North of Scotland study stated that a blog which posted views and experiences of current primary care trainees positively influenced their choice of location for primary care training [52].

### Mixed incentives

Weak evidence was found for mixed incentives. Five small studies [53–57] evaluated mixed incentives, combining continued medical education, financial and undergraduate placement incentives to recruit and retain doctors, with mixed results. None of the studies included a comparison group and therefore it is difficult to draw conclusions about either individual components or the intervention as a whole.

Two of these studies evaluated one scheme in Alberta, which used financial incentives, CME and rotations aimed at undergraduates, postgraduates and currently practicing doctors [56, 57] 35 % indicated the scheme had a critical or moderate effect on their decision to move or stay in Alberta but after the scheme was initiated the number of rural primary care doctors in Alberta actually reduced [56].

### Support for professional development and academic opportunities

Five studies, without a comparison group, focused on interventions which aimed to provide primary care doctors with an increase in academic skills, particularly in teaching and research [58–62]. Three studies reported the London Initiative Zone Educational Incentive scheme (LIZEI) aimed to improve recruitment, retention and refreshment of London GPs via various schemes which focused on increasing the academic aspect of training through academic/research associate schemes [58–60]. The scheme reported high levels of retention (75 % from the London Academic Training Scheme (LATS) cohort continued to practice in London) [58], but the lack of a comparison group makes this difficult to interpret, two further studies echoed this result, and found high levels of retention in London [59, 60]. The two other studies were from Australia [61, 62]. One found that 80 % of attendees (341/426) of continuing medical education (CME) workshops reported that they were less likely to remain in rural practice without CME [62]. The other study lack sufficient detail to allow interpretation of the results [61].

### Retainer schemes

Four studies, without a comparison group, assessed retainer schemes in the UK, including the Women’s Doctors Retainer Scheme [63, 64], the GP retainer scheme [65] and the Doctors’ retainer scheme [66]. Retainer schemes allow primary care doctors to work reduced hours (a maximum of four sessions a week, a minimum of one) with an educational component, for up to five years. Participants from a retainer scheme in Scotland reported that the scheme prevented them leaving medicine (32 % of former members (33/104) and 46 % of current members (69/152) [66]. All studies showed high retention of primary care doctors from retainer schemes (86 % (91/105) [65], 91 % (33/36) [63] and 71 % (10/14) [64] but the lack of a comparator group made it difficult to draw conclusions about the effect of the scheme.

### Re-entry schemes

One small study [67] with a comparison group evaluated a re-entry scheme, developed to help doctors to return to general practice as a partner (i.e. partner is a term used in the UK for a GP who makes a financial investment into a practice, and can therefore benefits from any profits (or losses) it makes, they must also oversee how the practice is run). The scheme rebuilt their confidence using needs based tutorials. Six months after the course 2 out of 14 attendees had returned to working as partners. 11 out of 14 attendees had taken ‘positive steps’ (this was not explained further) to return to general practice or had increased their time commitment to medicine. Compared with 1 in the control group (comparison group denominator not reported), who had ‘made plans’ to return to general practice [67]. The numbers were too small to draw conclusions.

### Delayed partnership

Weak evidence was found for the value of delayed partnerships. Two studies without comparison groups looked at delaying partnership after GP training by adding up to two years of post-vocational training [68, 69]. This included sessions at a mentor practice gaining general experience, varied locum experience and protected time for further training education [68]. Another scheme added one year of extra training which included exposure to the financial, managerial aspects of partnership, as well as clinical time [69]. The lack of a comparison group made it impossible to draw conclusion about the effectiveness of delayed partnerships.

### Specialised recruiter or case managers

The weakest evidence was found for specialised recruiters or case managers. Two cross sectional studies (non-comparative) used specialised recruiters or case managers to recruit doctors to rural areas [70, 71]. They provided a holistic approach to recruitment, identifying any particular needs of the doctor, helping to support them through the transition and encouraging community development activities. While both studies reported successful recruitment (17 doctors in 18 months [71] and 8 primary care providers in two years [70]), the impact of a case manager is unclear without a comparison group. A bachelor’s degree in a health related field plus health related work experience (two years minimum) was required for the specialised recruiter post in the USA, but was not compulsory [70].

## Discussion

This is the first systematic review to assess interventions to improve recruitment and retention of primary care doctors. The studies were all of low methodological quality, and only 15 of the 51 included studies involved a comparison group. There is weak evidence from these 15 studies that improved recruitment of primary care doctors was associated with postgraduate placements in underserved areas, undergraduate rural placements and recruiting students to medical school from rural areas. There was weak mixed evidence about financial incentives. The quality of the studies was not sufficient to draw conclusions about retainer schemes, re-entry schemes, international recruitment, specialised recruiters, support for professional development or research, delayed partnerships, well-being or peer support or mixed approaches.

### Strengths and limitations

Strengths of this review included that an inclusive search was undertaken with a robust process for screening and extraction of data. Grey literature was extensively searched and yielded six additional studies. Authors were contacted when necessary and this resulted in two additional studies. The methodological quality was assessed using a modified Newcastle Ottawa Scale, to elicit particular methodological problems. The response rate for the questionnaire based studies varied between 55 and 100 %. An online survey achieved a 100 % (24/24) response rate [46].

The methodological quality of the included studies was low as there were no RCTs and many of the studies did not include a control group or comparator. 21/51 of the included studies had some conflict of interest.

Studies without a defined intervention were excluded, this may have restricted our search however we wanted to evaluate how well interventions worked, not simply find relationships or factors which influence recruitment and retention. In eight studies the outcome was the self-reported location of where the trainee or doctor was practicing after the intervention, which may be open to reporting bias.

Sample sizes were generally small and 7 studies had less than 20 participants, which may affect the generalisability of the results. Some studies may have been affected by selection bias as for example students with an interest in rural medicine and intentions to continue with it may be more likely to join a rural based university or program.

Many of the interventions were tested in the USA, Canada or rural Australia. Whilst this may limit generalisability to other countries and health systems, the principles and theory behind the interventions such as providing placements in underserved areas should be considered for undergraduate and postgraduate training for primary care may be transferable. There was great variability in reported outcome measures and ‘retention rates’. Some studies regarded the intention to stay in the area after an intervention as retention, whilst others considered the length of duration post- intervention as retention. The heterogeneity of outcome measure made comparison between studies difficult and meta-analysis impossible.

### Comparison with previous research

Previous systematic reviews of strategies to increase attraction and retention of health workers in rural areas found some evidence to support the use of financial incentives, and insufficient evidence for increasing professional support, support for medical education and educational initiatives [72, 73]. A systematic review of recruitment and retention of primary care doctors in rural Canada and Australia found that factors before medical school were associated with future practice location, and that those who had been bought up or completed high school in rural areas were subsequently more likely to work there [72]. Rural experiences in postgraduate training, financial incentives, and support for professional development were found to be valuable [72].

### Comparison of our findings with existing recommendations

In the UK the 10 Point Plan [15] plans to introduce an additional flexible year, after completion of training to recruit trainees to underserved areas is planned [15]. This was supported by our research which found that doctors who completed their training in underserved areas were statistically significantly more likely to practice there. The report also sets out a plan to explore a three year financial incentive scheme to offer additional financial support to GP trainees committed to working in underserved areas [15]. Our research found mixed results for the use of financial incentives. They were found to be particularly successful when tying in trainees who had existing links to the underserved area [21], and when there is a long period of service obligation and more flexibility in career opportunities [21, 22, 62]. Financial incentives may need to be tied into public sector service with clear guards against direct personal profit.

A new induction and refresher scheme has been recently introduced in the UK by Health Education England and aims to support GPs to return to the workforce in England [16]. The GP taskforce final report recommends funding of a returners scheme – prioritising funding in under-doctored areas [4]. Our research did not find sufficient evidence to support or refute this. A marketing campaign is set to be implemented (a recruitment video outlining the positive aspects of general practice has already been distributed by RCGP). Our research found that video marketing may have a negative effect on recruitment.

### Implications for research and policy and conclusions

Despite the large number of reports and studies on the primary care doctors workforce crisis, and papers describing factors which influence recruitment and retention there is little evidence about which interventions are actually effective. As is pointed out in the recommendations of the Roland Commission [74], policy makers and health planners cannot learn from previous initiatives without published high quality evaluation, and unsuccessful strategies risk being reintroduced repeatedly, and consequently substantive amounts of funding wasted.

The evidence from this review also suggests that selection and educational exposures are important and that students are likely to be retained in a rural or underserved area if they have connections to the area, and are exposed to good educational primary care placements. Despite the recruitment crisis, clinical time as an undergraduate remains dominated by hospitals in many UK medical schools, and there is a great disparity between the numbers of graduates who eventually training as GPs. The 2015 F2 Career Destination Report shows that some UK universities are producing significantly more graduates who go on and apply to GP training than others, with Oxford and Cambridge behind others (see Table 3) [75]. Medical schools have a responsibility to start taking notice of the workforce crisis in primary care and perhaps resources and funding for these Universities should be based on output to meet targets. Furthermore if the UK is to meet the targets of 50 % of medical students entering primary care, we must consider more medical school training to be in GP and resources to support this must be provided. Universities should be held accountable as to how much time they allocate to the primary care setting and this data should be made publically available. The current work plan for primary care doctors is outdated, there is a need for change to reflect the change in the workforce. As the recruitment crisis worsens we believe these methods should be trialled on a larger scale.

**Table 3:** The table below shows the percentage of respondents who were appointed to GP specialty training in the UK from each UK foundation school

Rank	UK Medical School	% appointed to GP training in UK
1	Lancaster School of Health and Medicine	30.0 %
2	Keele University	29.5 %
3	University of Aberdeen	26.9 %
4	University of East Anglia	26.6 %
5	University of Leicester	26.4 %
6	Hull York Medical School	26.3 %
7	Queen Mary University of London	25.7 %
8	University of Warwick	24.7 %
9	St Georges, University of London	22.2 %
10	The University of Sheffield	21.6 %
11	Cardiff University	20.5 %
12	University of Leeds	19.2 %
13	Kings College	19.1 %
14	University of Liverpool	18.9 %
15	University of Dundee, Faculty of Medicine	18.2 %
16	Peninsula College of Medicine and Dentistry	17.5 %
17	University of Glasgow	17.5 %
18	University of Birmingham	16.8 %
19	University of Nottingham	15.7 %
20	Brighton and Sussex Medical School	15.6 %
21	Imperial College School of Medicine	15.4 %
22	University of Manchester	13.0 %
23	University of Newcastle	12.6 %
24	University of Bristol	11.3 %
25	University College London	10.8 %
26	Queens University Belfast	10.6 %
27	The University of Southampton	10.5 %
28	The University of Edinburgh	10.4 %
29	University of Oxford	9.2 %
30	University of Cambridge	7.3 %

More research is needed to identify evidence-based solutions to the primary care doctor workforce crisis and specific interventions to encourage more doctors into deprived areas that are currently under-served. Evaluations should be designed into all new recruitment initiatives and should include a comparison group (e.g. before and after, multicentre comparative, or formal trial) and these should then be made publically available. Retention based initiatives/interventions must also be tested to help streamline the current process of qualified GPs returning to work after a career break, and to help aid the loss of doctors to overseas positions, or those choosing to leave the profession entirely. Novel theory-based strategies such as case managers, improved support for professional development, and well-being and peer support should be evaluated.

## Conclusions

Although the evidence base for recruiting and retaining primary care doctors is weak and more high quality research is needed, this review found evidence to support undergraduate and postgraduate placements in underserved areas, and selective recruitment of medical students. The other initiatives covered in this review all have potential to improve recruitment and retention of primary care practitioners, but their effectiveness is not yet established.

## Ethical approval

None required. No primary data collection.

## Consent for publication

Not applicable.

## Availability of data

No additional data available.

## Appendix 1

### MEDLINE search strategy

1. exp physician/
2. exp general practice/
3. general practitioner*.tw.
4. (family adj doctor*).tw.
5. GP*.tw.
6. general practitioner/
7. physicians, family/
8. physicians, primary care/
9. doctor*.tw.
10. 1 or 2 or 3 or 4 or 5 or 6 or 7 or 8 or 9
11. exp personnel selection/
12. (recruit* adj2 (GP* or doctor* or (medical adj (personnel or staff or professional* or worker*)) or (General adj practitioner*) or (family adj physician*) or (family adj doctor*))).tw.
13. (retain* adj2 (GP* or doctor* or (medical adj (personnel or staff or professional* or worker*)) or (General adj practitioner*) or (family adj physician*) or (family adj doctor*))).tw.
14. (retention* adj2 (GP* or doctor* or (medical adj (personnel or staff or professional* or worker*)) or (General adj practitioner*) or (family adj physician*) or (family adj doctor*))).tw.
15. 11 or 12 or 13 or 14
16. 10 and 15
17. limit 16 to humans

### Search strategy EMBASE 3/12/14

1. exp physician/
2. exp general practice/
3. general practitioner*.tw.
4. (family adj doctor*).tw.
5. GP*.tw.
6. general practitioner/
7. physicians, family/
8. physicians, primary care/
9. doctor*.tw.
10. 1 or 2 or 3 or 4 or 5 or 6 or 7 or 8 or 9
11. (recruit* adj2 (GP* or doctor* or (medical adj (personnel or staff or professional* or worker*)) or (General adj practitioner*) or (family adj physician*) or (family adj doctor*))).tw.
12. (retain* adj2 (GP* or doctor* or (medical adj (personnel or staff or professional* or worker*)) or (General adj practitioner*) or (family adj physician*) or (family adj doctor*))).tw.
13. (retention* adj2 (GP* or doctor* or (medical adj (personnel or staff or professional* or worker*)) or (General adj practitioner*) or (family adj physician*) or (family adj doctor*))).tw.
14. *personnel management/
15. 11 or 12 or 13 or 14
16. 10 and 15
17. limit 16 to human

## Abbreviations

AS: Arabella Stuart
CME: continuing medical education
GP: general practitioner
IMG: international medical graduates
JF: John Ford
LATS: London Academic Training Scheme
LIZEI: London Initiative Zone Educational Incentive Scheme
NHS: National Health Service
NHSC: National Health Service Corps
NS: Nick Steel
PV: Puja Verma
RCGP: Royal College of General Practitioners
RCT: randomised controlled trial
UK: United Kingdom
USA: United States of America
WHO: World Health Organisation
